# Direct Inhibitory Effects of Pioglitazone on Hepatic Fetuin-A Expression

**DOI:** 10.1371/journal.pone.0088704

**Published:** 2014-02-13

**Authors:** Akinobu Ochi, Katsuhito Mori, Masanori Emoto, Shinya Nakatani, Tomoaki Morioka, Koka Motoyama, Shinya Fukumoto, Yasuo Imanishi, Hidenori Koyama, Eiji Ishimura, Masaaki Inaba

**Affiliations:** 1 Department of Metabolism, Endocrinology and Molecular Medicine, Osaka City University Graduate School of Medicine, Osaka, Japan; 2 Department of Internal Medicine, Division of Endocrinology and Metabolism, Hyogo College of Medicine, Nishinomiya, Japan; Clermont Université, France

## Abstract

Fetuin-A, a circulating glycoprotein synthesized in the liver, is involved in insulin resistance and type 2 diabetes. However, regulation of fetuin-A synthesis has remained obscure. We previously reported that pioglitazone treatment significantly reduced serum fetuin-A levels in patients with type 2 diabetes. To clarify whether pioglitazone can directory inhibit hepatic fetuin-A synthesis, we investigated the effects of pioglitazone on fetuin-A expression both in vitro and in vivo. Pioglitazone treatment suppressed mRNA and protein expression of fetuin-A in Fao hepatoma cells. Interestingly, rosiglitazone but not metformin, also inhibited fetuin-A expression. In addition, GW 9662, an inhibitor of peroxisome proliferator-activated receptor (PPAR) γ, reversed pioglitazone-induced suppression of fetuin-A, suggesting that thiazolidinedione derivatives may have common characteristics with regard to fetuin-A suppression, possibly through PPARγactivation. Finally, oral administration of pioglitazone to mice for 8 weeks resulted in suppression of hepatic fetuin-A mRNA. These findings suggest that pioglitazone may partially ameliorate insulin resistance through its direct inhibitory effects on fetuin-A expression in the liver.

## Introduction

Fetuin-A (alpha-2-HS-glycoprotein; AHSG) is mainly synthesized in the liver and has multiple functions in human physiology and pathology [1], [2]. Among them, fetuin-A known as one of most important hepatokines [3] is involved in insulin resistance and type 2 diabetes, although its precise role is poorly understood. Cross-sectional studies have demonstrated a positive correlation between fetuin-A levels and insulin resistance [4]. Prospective studies also have shown that high fetuin-A levels predict the incidence of type 2 diabetes [5] [6]. As one of its mechanisms, both in vitro and in vivo studies have suggested a direct inhibitory effect of fetuin-A on insulin receptor autophosphorylation, which alters downstream signaling [7]. In addition, a recent report has made a breakthrough in this area. It is well known that free fatty acids (FFAs) stimulate secretion of proinflammatory cytokines from adipocytes through Toll-like receptor 4 (TLR4), resulting in insulin resistance [8]. However, it remained obscure how FFAs can induce adipose tissue inflammation, since FFAs appear to not bind to TLR4 directly [9]. Facing such criticism, Pal and Dasgupta et al. clearly demonstrated that fetuin-A acts as an adaptor protein between FFAs and TLR4 [10]. These findings suggest that fetuin-A may amplify insulin resistance through this pathway. In response, Stefan et al. reported the probability that an interaction between FFAs and fetuin-A could induce insulin resistance in humans [11]. Therefore, although the regulation of fetuin-A synthesis is incompletely understood, fetuin-A is an attractive target for the development of novel diabetes treatments.

Pioglitazone, one of thiazolidinedione derivatives, is an established insulin-sensitizing agent used by patients with type 2 diabetes. Its unique action is predominantly exerted through peroxisome proliferator-activated receptor (PPAR) γin adipocytes [12]. We previously reported that treatment with pioglitazone significantly reduced serum fetuin-A levels in patients with type 2 diabetes [13]. Intriguingly, other representative insulin-sensitizing therapies, such as metformin (500 mg/day or 750 mg/day) and aerobic exercise, did not affect fetuin-A levels [13], although it has been reported that high dose of metformin (2500 mg/day or 3000 mg/day) decreased plasma fetuin-A levels [14]. Therefore, fetuin-A-mediated improvement of insulin resistance is likely to be a particular property of pioglitazone. However, in the previous clinical study we could not conclude whether pioglitazone directly inhibited fetuin-A synthesis in the liver because the pleiotropic effects of pioglitazone on glucose and lipid metabolism [12] may have indirectly modulated fetuin-A levels.

To clarify the direct inhibitory effect of pioglitazone on fetuin-A synthesis, we examined the effects of pioglitazone on fetuin-A expression in cultured hepatocytes. We also investigated whether administration of pioglitazone to mice could suppress fetuin-A expression in the liver, resulting in decreased circulating fetuin-A levels.

## Materials and Methods

### Reagents

All cell culture plasticware was purchased from Becton, Dickinson and Company (NJ, USA). Pioglitazone hydrochloride, rosiglitazone, glimepiride, GW9662, and dimethyl sulfoxide (DMSO) were obtained from Wako Pure Chemical Industries Ltd. (Osaka, Japan). Metformin (1,1-dimethyl biguanide hydrochloride) was purchased from Sigma Aldrich (MO, USA).

### Cell Culture

Rat hepatoma cells (Fao cells) were provided courtesy by Dr. Rohit N. Kulkarni (Joslin Diabetes Center, Boston, MA) [15]. Cells were cultured and maintained in RPMI-1640 medium (Invitrogen, CA, USA) supplemented with 10% heat-inactivated FBS (Invitrogen), 100 U/mL penicillin, and 100 µg/mL streptomycin (Invitrogen) in humidified air with 5% CO2 at 37°C [15]. The culture medium was replaced every 2 days. After cultures reached confluence, cells were serum-starved overnight and subsequently incubated for different periods (0, 6, 12, 18, and 24 h) and at different doses (0, 2, 5, and 10 mM) of pioglitazone. Similarly, cells were treated with or without rosiglitazone (10 µM), metformin (1 or 2 mM), or glimepiride (1, 5, or 10 µM) for 24 h. Cells were preincubated with GW9662 for 1 h before addition of pioglitazone.

### RNA Isolation and Real-time Quantitative RT-PCR

RNA [2 µg, isolated from cells by using TRIzol Reagent (Invitrogen)] was used to synthesize cDNA by reverse transcription with the High Capacity cDNA Reverse Transcription Kit (Applied Biosystems, CA, USA) according to manufacturer’s instructions. Expression of rat or mouse *Ahsg* mRNA, and 18S ribosomal RNA were measured quantitatively using the Taqman Real-Time PCR technique (Applied Biosystems). Ready-to-use primers for rat or mouse fetuin-A mRNA and 18S ribosomal RNA were purchased from Applied Biosystems (Taqman Gene Expression Assays) and used according to manufacturer’s instructions. A cycle threshold (Ct) value was measured for each sample, and mRNA expression levels were determined by a comparative Ct method using 18S ribosomal RNA as endogenous control (Applied Biosystems) [16].

### Western Blotting

After each treatment, the cells were washed twice with ice-cold buffer (137 mM NaCl, 1 mM MgCl_2_, 1 mM CaCl_2_, 0.1 mM Na_2_VO_4_, 20 mM Tris-HCl at pH 7.6) and lysed in the same buffer supplemented with 1% Nonidet P-40, 10% glycerol, 2 mM EDTA, 10 mM sodium pyrophosphate, 10 mM NaF, 2 mM Na_2_VO_4_, 2 mM phenylmethylsulfonyl fluoride, and 8 µg/mL leupeptin. After removal of insoluble materials, the protein concentration was determined using the BCA protein assay (Thermo Scientific, MI, USA). Cell lysates or culture supernatant medium (40 µg protein per lane) were mixed with a sample-loading buffer and separated on a 10% SDS-PAGE gel. Proteins were transferred to nitrocellulose membranes (GE Healthcare, Buckinghamshire, UK) and immunoblotted with rat fetuin-A antibody (Santa Cruz Biotechnology Inc, CA, USA) and α-tubulin antibody (Cell Signaling Technology, MA, USA) [17]. Protein bands were identified by ECL (GE Healthcare, NJ, USA) and quantified using a densitometer (Lumino Shot™ 400Jr) (TAKARA Bio Inc, Shiga, Japan).

### Animal Experiments

All procedures in this study were approved by the Animal Care and Use committee at the Osaka City University Graduate School of Medicine, Osaka, Japan [16]. Male C57BL/6J mice were maintained in a temperature-controlled (24°C) facility with a 12-h light/12-h dark cycle and fed a standard chow *ad libitum*. At 7 weeks, the mice were divided into a group bred with pioglitazone, delivered as a food admixture at 10 mg/kg/day, (Pio group: *n* = 6) and a group bred without pioglitazone (CTL group: *n* = 6). Mice were fed with the appropriate diet from 7 to 20 weeks of age. Body weight was monitored weekly. Blood glucose levels were also measured weekly using a portable glucose meter (Glu-test Sensor, Sanwa, Japan). At 20 weeks of age, mice were fasted overnight, and then sacrificed under the anesthesia with pentobarbital; liver tissues and blood samples were collected. Hepatic mRNA expression in CTL or Pio group was examined as mentioned above. Serum fetuin-A levels in CTL or Pio group were measured by enzyme-linked immunosorbent assay kit (Mouse fetuin-A/AHSG ELISA kit, Adipo Bioscience, CA, USA).

### Statistical Analysis

Each experiment was repeated at least thrice to confirm the results. All data are expressed as mean ± standard deviation (SD). Student’s t-test was used for comparisons between two groups. Statistically significant differences among more than three groups were analyzed using ANOVA with post hoc analysis (Dunnet or Scheffe test). All analyses were performed using StatView 5 (SAS Institute Inc., Cary, NC, USA) designed for Windows. The level of statistical significance was set at *P*<0.05.

## Results

### Fetuin-A Expression in Hepatocytes and its Posttranslational Modification

**Figure 1:**
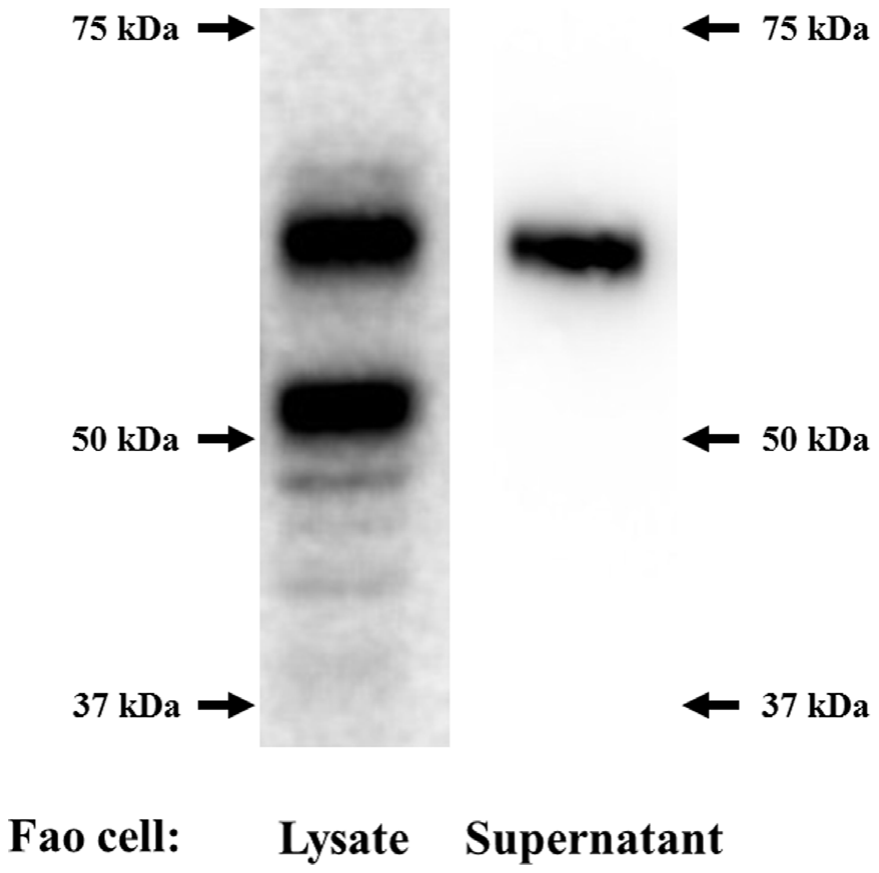
Fetuin-A expression in hepatocytes and its posttranslational modifications. Lysates from Fao cells (left panel) and the supernatant from cultured Fao cells (right panel) were run on a 10% SDS-PAGE gel, transferred to a membrane, and probed using an anti-fetuin-A antibody.

Fetuin-A is known to be posttranslationally modified, including serine and threonine phosphorylations and glycosylations [7]. Therefore, the apparent molecular weight of fetuin-A on an immunoblot is approximately 60 kDa, which is much higher than deduced molecular weight based on the amino acid sequence. We detected two primary bands using a cell lysate from Fao cells (Fig. 1, left panel). The lower band may correspond to the endogenous fetuin-A protein before apparent modification. To confirm this, we transfected HEK 293 cells, which are believed not to have the ability to perform liver-specific posttranslational modification, with mouse fetuin-A (*Ahsg*) cDNA, provided courtesy by Dr. Jiang (Thomas Jefferson University, PA, USA) [18]. As expected, we observed only one band, which migrated at approximately 50 kDa in the immunoblot (data not shown). Next, culture medium from Fao cells was subjected to immunoblotting for fetuin-A. Only the upper band (60 kDa) was detected, suggesting that only fully modified fetuin-A is secreted from hepatocytes (Fig. 1, right panel).

### Inhibitory Effects of Pioglitazone on both mRNA Expression and Protein Expression of Fetuin-A

**Figure 2:**
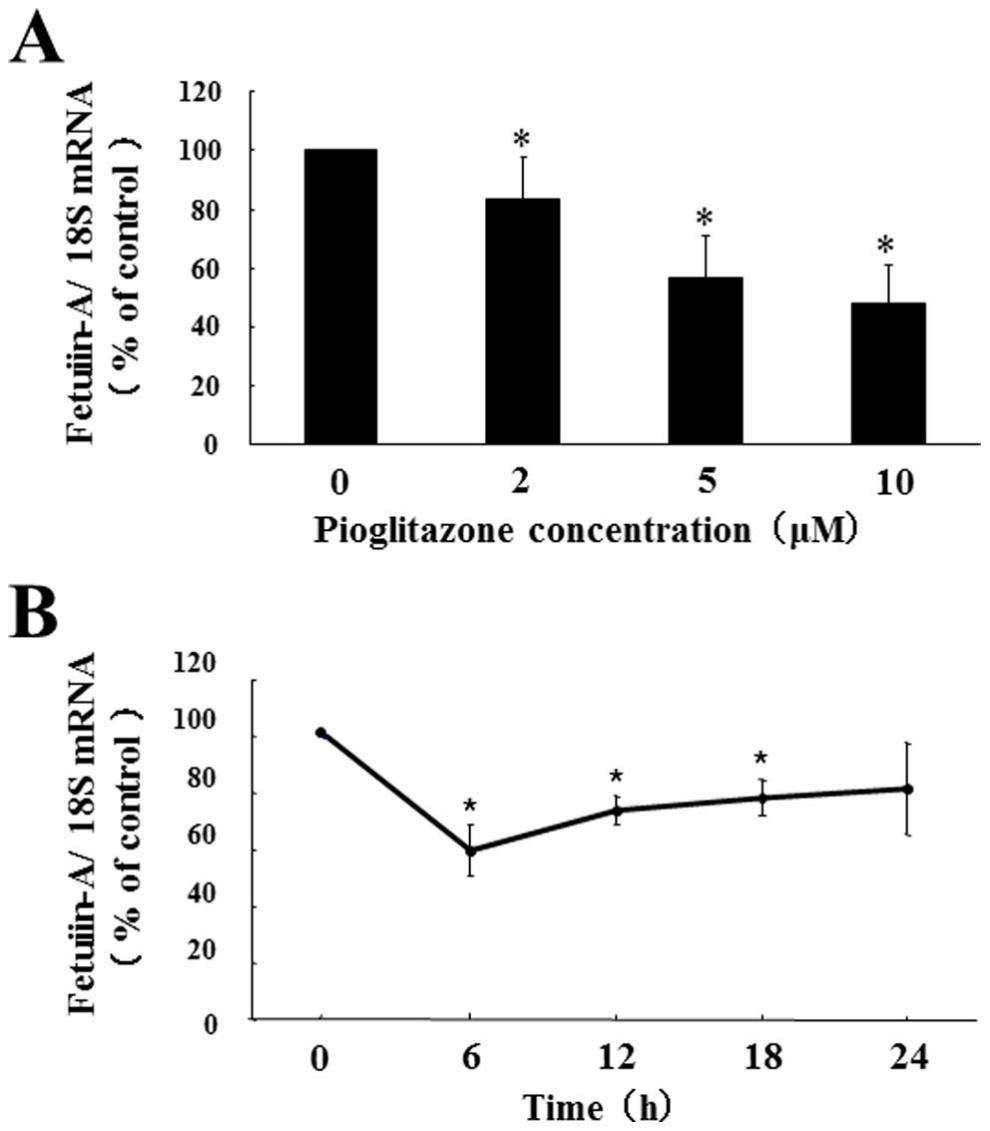
Effects of pioglitazone on expression of fetuin-A mRNA in Fao cells. Fao cells were treated with pioglitazone at different doses (0, 2, 5, and 10 µM for 24 h) (A) or for different periods (0, 6, 12, 18, and 24 h with 10 µM) (B). Total RNA was isolated from Fao cells treated with or without pioglitazone, and the levels of fetuin-A mRNA expression were determined using quantitative real-time RT-PCR. Levels of fetuin-A mRNA were determined using a comparative cycle threshold method, using 18S ribosomal RNA as endogenous reference, and fetuin-A mRNA levels without pioglitazone (A) or at 0 h (B) were defined as 100% (CTL). Mean ± SD were calculated from at least three independent experiments. *P<0.05 vs. CTL.

**Figure 3:**
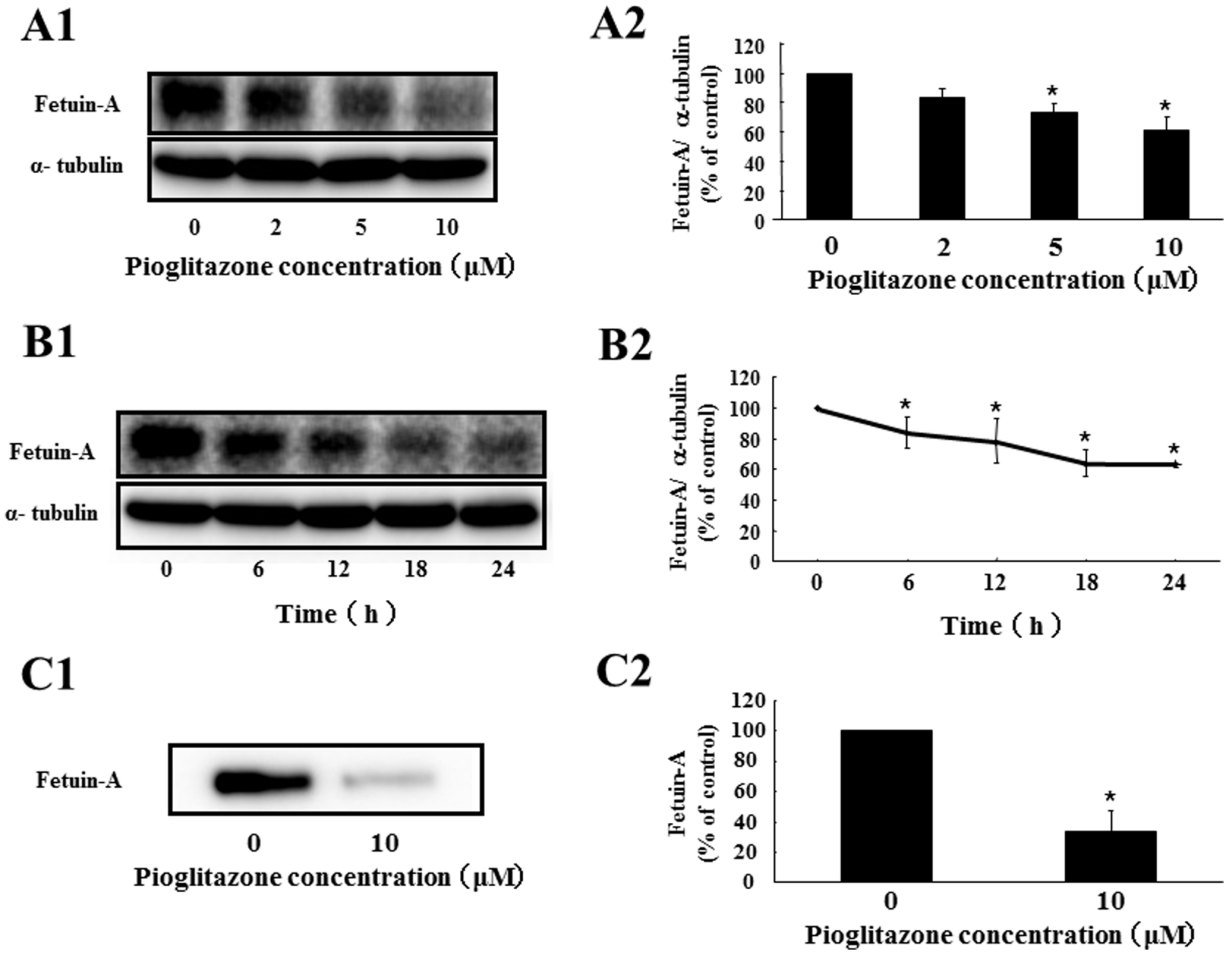
Effects of pioglitazone on fetuin-A protein expression in Fao cells. Fao cells were treated with pioglitazone at different doses (0, 2, 5, and 10 µM for 24 h) (A) or for different periods of time (0, 6, 12, 18, and 24 h with 10 µM) (B). Western blots of cell lysates from Fao cells treated with or without pioglitazone were probed with anti-fetuin-A antibody (A1) (B1). Fetuin-A protein levels were determined using α-tubulin as an endogenous reference, and fetuin-A levels without pioglitazone (A2) or at 0 h (B2) were defined as 100% (CTL). Western blots of Fao cell supernatants with or without 10 µM pioglitazone for 24 h were probed with anti-fetuin-A antibody (C1). Fetuin-A protein levels from experiments without pioglitazone were defined as 100% (CTL). Mean ± SD were calculated from at least three independent experiments. *P<0.05 vs. CTL.

To investigate whether pioglitazone can directly suppress fetuin-A expression in hepatocytes, we cultured Fao cells with or without pioglitazone. Pioglitazone downregulated fetuin-A mRNA expression (51.8% decrease by 10 µM pioglitazone for 24 h) in a dose-dependent manner (Fig. 2A). Pioglitazone (10 µM) significantly suppressed fetuin-A mRNA levels at 6 h (38.5% decrease) and maintained this suppression at 24 h (Fig. 2B). Next, we examined the effects of pioglitazone on fetuin-A protein expression in Fao cells and found that pioglitazone inhibited fetuin-A protein expression in dose- (Fig. 3 A1 and A2) and time- (Fig. 3 B1 and B2) dependent manner (38.8% decrease by 10 µM pioglitazone for 24 h). We also investigated whether pioglitazone decreased the levels of fetuin-A secreted into the cultured medium. As expected, pioglitazone strikingly suppressed fetuin-A secretion (66.3% decrease by 10 µM pioglitazone for 24 h) (Fig. 3 C1 and C2). These findings suggest that pioglitazone directly suppresses fetuin-A expression in heoatocytes and subsequently reduces secretion of posttranslationally modified fetuin-A.

### Specificity of Pioglitazone-induced Fetuin-A Suppression

**Figure 4:**
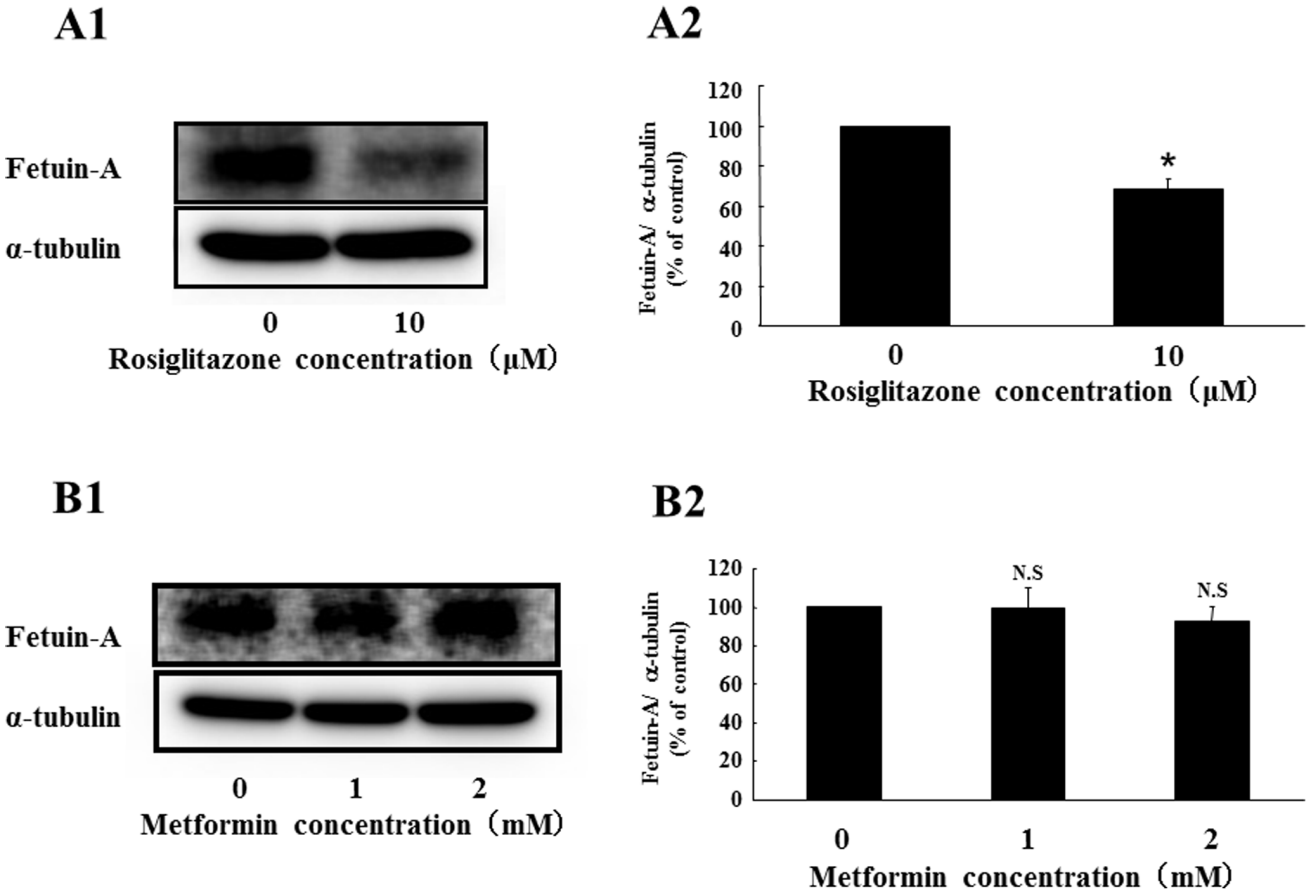
Effects of rosiglitazone or metformin on expression of fetuin-A protein in Fao cells. Fao cells were treated with rosiglitazone (0 and 10 µM) or metformin (0, 1, and 2 mM) for 24 h. Western blots of cell lysates from Fao cells treated without or with rosiglitazone or metformin were probed with anti-fetuin-A antibody (A1) (B1). Fetuin-A protein levels were determined using α-tubulin as an endogenous reference, and fetuin-A protein levels without rosiglitazne (A2) or metformin (B2) was defined as 100% (CTL). Mean ± SD were calculated from at least three independent experiments. *P<0.05 vs. CTL.

To examine the specificity of pioglitazone-induced fetuin-A suppression, we used rosiglitazone, another thiazolidinedione derivative. Rosiglitazone significantly suppressed fetuin-A protein expression (31.8% decrease by 10 µM rosiglitazone for 24 h) (Fig. 4A1 and 4A2). On the other hand, metformin, another insulin-sensitizing drug, did not affect fetuin-A expression (Fig. 4B1 and 4B2). In addition, the sulfonylurea glimepiride, a representative antidiabetic drug, had no effect on fetuin-A expression (data not shown). These findings suggest that pioglitazone-induced fetuin-A suppression may be a class effect of thiazolidinedione derivatives. However, it is not common in insulin-sensitizing drugs.

### Involvement of PPARγ in Pioglitazone-induced Suppression of fetuin-A

**Figure 5:**
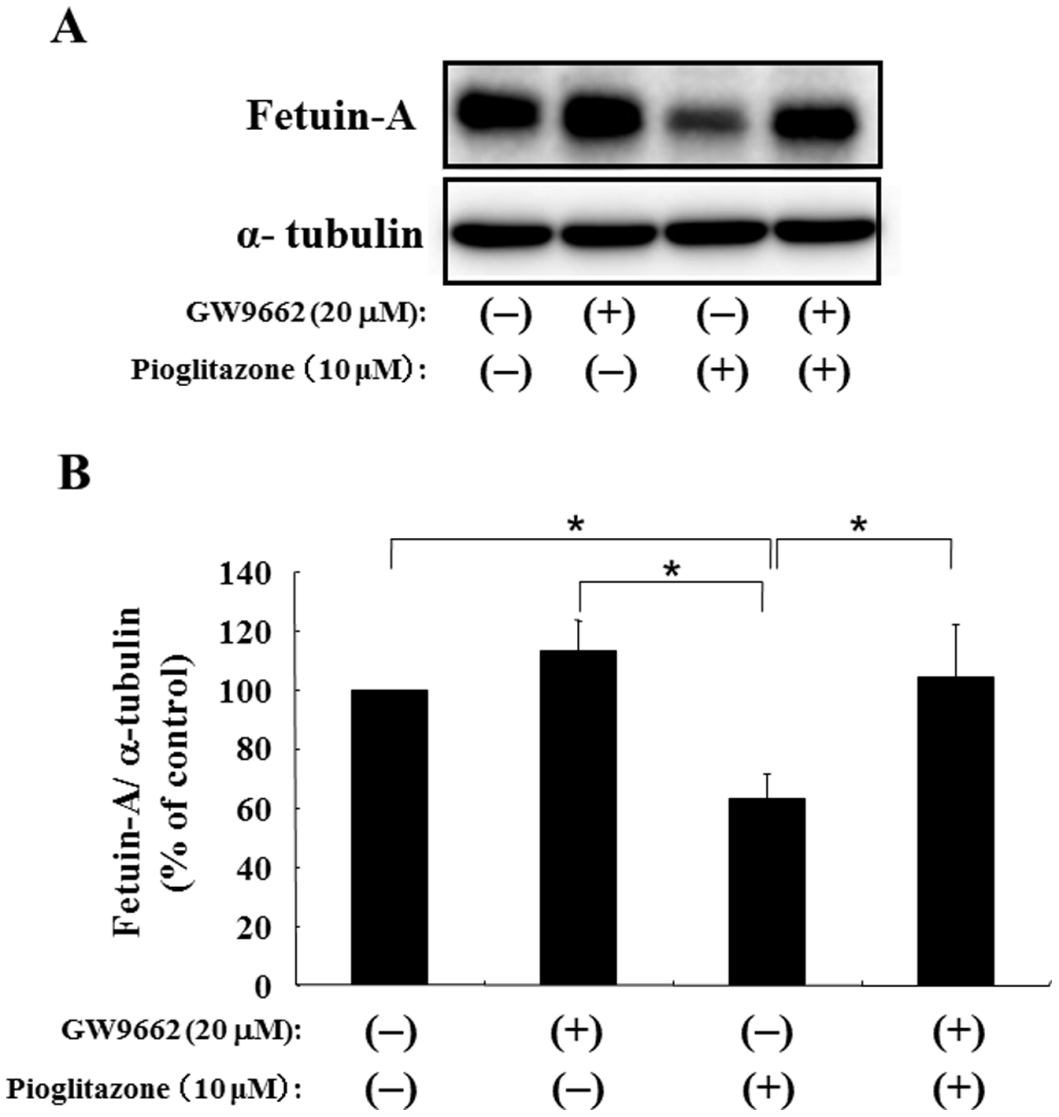
Effects of a PPARγ inhibitor (GW9662) on pioglitazone-induced suppression of fetuin-A in Fao cells. Fao cells were treated in the absence or presence of 10 µM pioglitazone for 24 h with or without 1-h preincubation with 20 µM GW9662, an inhibitor of PPARγ. Western blots of cell lysates from Fao cells were probed with anti-fetuin-A antibody (A). Fetuin-A protein levels were determined using α-tubulin as an endogenous reference, and the fetuin-A protein level without both pioglitazone and GW9662 was defined as 100% (CTL). Mean ± SD was calculated from at least three independent experiments. *P<0.05 between each group.

To have better insight into the mechanism, we investigated whether pioglitazone-induced suppression operated through PPARγ, a receptor for thiazolidinedione derivatives. GW9662, an inhibitor of PPARγ, did not change fetuin-A expression significantly (Fig. 5A). Interestingly, treatment with GW9662 reversed pioglitazone-induced suppression of fetuin-A to non-treatment levels (Fig. 5B). These findings suggest that pioglitazone suppresses fetuin-A expression though PPARγ.

### Effects of Pioglitazone on Hepatic Fetuin-A Expression and Serum Fetuin-A Level in Mice

**Figure 6:**
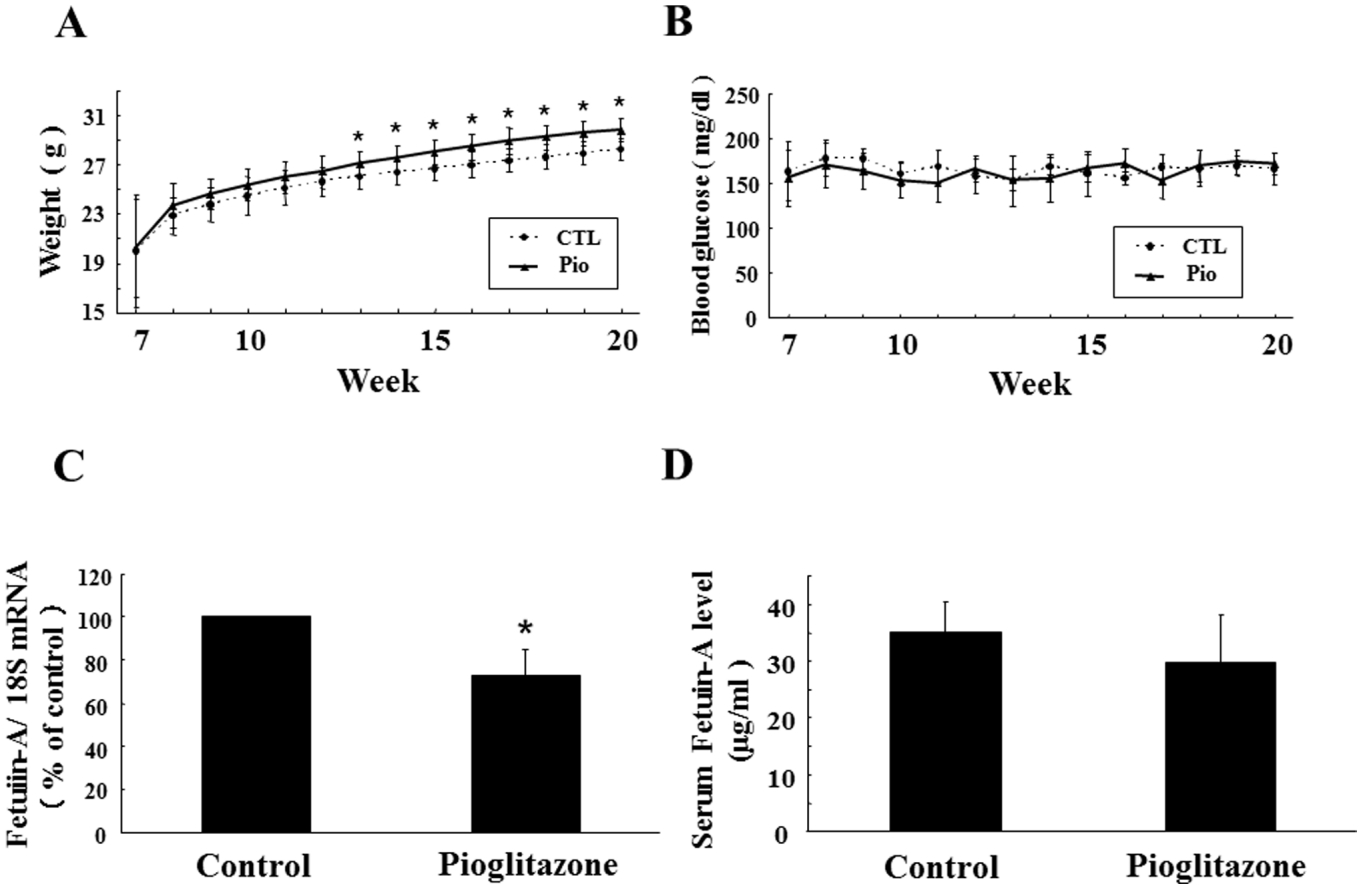
Effects of pioglitazone on hepatic fetuin-A expression and circulating fetuin-A levels in mice. Male C57BL/6J mice at 7 weeks of age were divided into a group bred with pioglitazone delivered as a food mixture at 10 mg/kg/day (Pio group: *n* = 6) and a group without pioglitazone (CTL group: *n* = 6). Body weight (A) and casual blood glucose levels (B) were monitored weekly in mice in each group from 7 weeks of age until 20 weeks of age. At 20 weeks of age, mice were sacrificed and liver tissues and blood samples were collected. Total RNA was isolated from mice in each group. Fetuin-A mRNA expression was determined using quantitative real-time RT-PCR. Fetuin-A mRNA expression levels were determined using a comparative cycle threshold method, with 18S ribosomal RNA as anendogenous reference. The fetuin-A mRNA level in the CTL group was defined as 100% (C). Circulating fetuin-A levels in the CTL- or Pio group were measured using an ELISA kit (D). Columns represented mean ± SD. *P<0.05 vs. CTL.

To minimize the impact of metabolic disorders, such as diabetes, dyslipidemia, and hepatic steatosis, on fetuin-A levels, we administered pioglitazone to healthy mice and investigated its effects on hepatic fetuin-A expression and circulating fetuin-A levels in vivo. Pioglitazone treatment induced significant body weight gain from week 13 onward (Fig. 6 A1), but did not affect casual plasma glucose levels (Fig. 6 A2). Importantly, hepatic expression of fetuin-A mRNA in the Pio group was significantly suppressed compared with that in the CTL group (27.1% inhibition, *P*<0.05). On the other hand, although serum levels of fetuin-A in the Pio group tended to be lower than those in CTL group, the difference did not reach statistical significance (29.7 vs. 35.3 µg/mL, *P* = 0.253).

## Discussion

Because of the critical role of fetuin-A in insulin resistance and type 2 diabetes, it is imperative to clarify how fetuin-A synthesis is regulated. We previously reported that pioglitazone, but not metformin, reduced serum fetuin-A levels in patients with type 2 diabetes [13]. However, its regulatory mechanisms have been unclear. In this study, we first demonstrated the direct inhibitory effect of piogliazone on fetuin-A expression, through PPARγ activation, in cultured hepatocytes. We also found that piogliazone treatment significantly inhibited hepatic fetuin-A expression in mice.

Considering the pleiotropic effects of pioglitazone, several possibilities remain with regard to pioglitazone-induced suppression of fetuin-A. First, the involvement of hepatic steatosis should be considered because circulating fetuin-A levels were positively associated with liver fat content evaluated by proton magnetic resonance spectroscopy in humans [19]. In addition, lifestyle intervention decreased both circulating fetuin-A levels and fat accumulation in the liver [19]. Interestingly, pioglitazone, but not metformin, could improve hepatic lipid content [20]. When taken together, pioglitazone can ameliorate hepatic steatosis, resulting in decreased circulating fetuin-A levels. Second, it has been reported that FFAs enhance fetuin-A expression in hepatocytes [21]. Pioglitazone is known to improve dyslipidemia, including high levels of FFAs [12]. Therefore, pioglitazone may decrease serum fetuin-A levels through reduction of FFAs. Finally, alteration of the adipokine profile caused by pioglitazone treatment could change fetuin-A levels. In this case, pioglitazone would act mainly on adipocytes through PPARγ, leading to upregulation of adiponectin, a representative adipokine [22]. Because adiponectin has been reported to alleviate fatty liver disease [23], pioglitazone may suppress fetuin-A levels by increasing adiponectin levels. All the speculations mentioned above describe indirect effects of pioglitazone on fetuin-A suppression. However, our findings suggest that pioglitazone can exert direct suppression of hepatic fetuin-A expression, though pioglitazone-induced indirect effects also could modify fetuin-A levels in vivo.

In one of its many roles, fetuin-A is well known to be a powerful calcification inhibitor [1], [2]. Its physiological role in the mortality of hemodialysis patients, its significance as an inhibitor of vascular calcification [24], and the techniques required to protect patients from ectopic calcification [25] have been established in detail. On the other hand, many clinical studies suggest that fetuin-A is involved in metabolic disorders, including diabetes and metabolic syndrome. Although fetuin-A can directly inhibit insulin receptor signaling in vitro [7], it has been unclear how fetuin-A acts on glucose and lipid metabolism with major force in vivo. Though we previously observed that pioglitazone treatment for 6 months reduced fetuin-A levels by approximately 13% [13], it has been a large open question whether such a slight alteration in fetuin-A, which is a very abundant serum protein, has a critical impact on systemic metabolism. Recently, Pal and Dasgupta et al. shed light on this deep question [10]. They proved that fetuin-A is necessary for lipid-induced chronic inflammation in adipose tissue, which is profoundly involved in metabolic disorders, including insulin resistance. FFAs activate intracellular NF-κB-associated genes, which encode inflammatory cytokines such as IL-6 and TNF-α through TLR4. In this process, fetuin-A functions as an adaptor between FFAs and TLR4 [10]. Therefore, small alterations in fetuin-A levels may lead to a substantial impact on metabolism through amplification mechanisms mediated by inflammatory cytokines. To confirm that these findings are relevant to the pathogenesis of insulin resistance in humans, Stefan et al. investigated the relationships between insulin resistance and circulating levels of fetuin-A and FFAs. They found a significant association between fetuin-A and insulin resistance in subjects with high FFAs but not in subjects with low FFAs. The positive association between FFAs and insulin resistance in subjects with high fetuin-A but not with low fetuin-A was also observed, suggesting that an interaction of FFAs with fetuin-A induces insulin resistance in humans [11].

PPARγ is predominantly expressed in adipocytes; its expression in liver is low. However, hepatic PPARγ plays a critical role in hepatic steatosis and in whole-body lipid and glucose homeostasis [26]. In this study, we found significant suppression of hepatic fetuin-A expression. Nevertheless, circulating fetuin-A levels were not significantly decreased in the pioglitazone-treated group compared with the control group. One possibility may be a relative small sample size (*n* = 6 in each group). Another explanation could be the condition of mice. To exclude the influence of metabolic disorders on fetuin-A expression, we chose healthy mice. Therefore, we may find an apparent reduction in circulating fetuin-A levels if we perform similar experiments in diabetic model mice because hepatic PPARγ expression is elevated in rodent models of diabetes or obesity [27].

With regard to the effect of metformin on fetuin-A expression, there seems to be inconsistent findings. Haukeland et al. found that metformin treatment for 6 months decreased plasma fetuin-A levels in patients with non-alcoholic fatty liver disease [14]. On the other hand, we found no changes in serum fetuin-A levels after intervention by metformin for 6 months in patients with type 2 diabetes [13]. However, the seemingly contradictory findings may be derived from the different dosages of metformin (500 mg/day or 750 mg/day [13] vs. 2500 mg/day or 3000 mg/day [14]). Similarly, Haukeland et al. reported that fetuin-A secretion from HepG2 cells decreased in the presence of metformin in a dose-dependent manner [14]. Again, the different amounts of metformin might lead to inconsistent results in in vitro studies. In fact, we detected the decrease of fetuin-A expression by treatment with high dose of 5 mM metformin in Fao cells (data not shown). Apart from this, it has been reported that high glucose enhanced fetuin-A promoter activity in HepG2 cells through activation of the ERK 1/2 signaling pathway, resulting in an increase of fetuin-A protein expression [28]. Therefore, to evaluate fetuin-A levels in patients with diabetes, glycemic levels should be also considered in addition to drug intervention.

In conclusion, pioglitazone can directly suppress hepatic fetuin-A expression both in vitro and in vivo. This appears to be a unique characteristic of thiazolidinedione derivatives compared with other antidiabetic drugs. Because emerging evidence suggests that fetuin-A may serve as a therapeutic target for metabolic disorders, including insulin resistance and type 2 diabetes, discovery of a liver-specific PPARγ agonist will provide the potential for an entirely new approach to treating metabolic disorders through fetuin-A regulation.
